# Ferric Carboxymaltose as an Effective and Safe Alternative to Iron Sucrose for Treatment of Iron Deficiency Anemia during Pregnancy

**DOI:** 10.34763/jmotherandchild.20252901.d-25-00018

**Published:** 2025-11-05

**Authors:** Aparajita Singh, Manu Goyal, Shashank Shekhar, Pratibha Singh, Praveen Sharma

**Affiliations:** Department of Obstetrics and Gynecology, All India Institute of Medical Sciences, Jodhpur, India; Department of Biochemistry, All India Institute of Medical Sciences, Jodhpur, India

**Keywords:** anemia, carboxymaltose, iron sucrose, pregnancy, efficacy

## Abstract

**Background:**

This was a prospective observational study to compare efficacy and safety of iron sucrose (FeS) and ferric carboxymaltose (FCM) in pregnancy conducted over 18 months at a tertiary hospital.

**Methods:**

Pregnant women between 14 to 36 weeks gestation with moderate to severe iron deficiency anemia were enrolled in the study. The primary outcome was a rise in haemoglobin after 14 and 28 days. Change in red cell indices, serum iron studies, symptomatic improvement, adverse effects, and neonatal outcomes were also compared.

**Results:**

95 pregnant women with anemia were included in the study. Mean rise in haemoglobin after 14 days was significantly higher in the FeS group than in the FCM group (2.25 ± 0.91 g/dL vs. 1.69 ± 0.98 g/dL, p value = 0.01), but rise in median serum ferritin was significantly more in the FCM group (148.55 vs. 310.6; p < .001). No significant adverse effect was noted in any group.

**Conclusion:**

Injectable iron preparation FeS results in an early rise in haemoglobin, while FCM leads to a higher rise in iron stores as seen by a rise in serum ferritin. As FCM requires fewer hospital visits, it is more convenient to the patient.

## Introduction

Anemia is one of the major causes of mortality and morbidity during pregnancy in the world. The World Health Organization (WHO) defines anemia in pregnancy as a haemoglobin concentration of less than 11 g/dL and hematocrit less than 33%. The Centers for Disease Control defines anemia as pregnancy haemoglobin of less than 11 g/dL (hematocrit < 33%) in the first and third trimester and less than 10.5 g/dL (Hematocrit < 32%) in the second trimester [1]. According to WHO, it can be attributed to 20% of the cases of maternal mortality [2]. According to the National Family Health Survey–4, the prevalence of anemia in India was 50.4% [3]. In developing countries, iron deficiency is the most common cause of anemia, both in the pregnant and non-pregnant state. It is a major concern, as iron deficiency during pregnancy poses great risk to both mother and fetus. Antenatal complications include increased risk of preterm labor, premature rupture of membranes, pre-eclampsia, intrauterine death, intercurrent infections, and congestive heart failure. Intrapartum complications include dysfunctional labor, obstetric haemorrhage, congestive heart failure, shock, fetal distress, and anaesthesia risk. Postpartum complications include haemorrhage, puerperal sepsis, subinvolution, lactation failure, and pulmonary thromboembolism. Tandon (2018) mentioned the potential fetal complications as growth restriction, prematurity, infections, low iron stores at birth, and abnormal cognitive development [4]. McDonagh (2015), in a systematic review, recommended screening of all antenatal women for anemia at the booking visit and then in every trimester [5].

Approximately 1000 mg of iron is required in a normal pregnancy, out of which 300 mg is transferred to the fetus and placenta, 500 mg for erythropoiesis; 200 mg is lost through physiological routes. When iron stores are low, the fetus gets priority, followed by mother’s hematocrit and then maternal stores. If the mother has inadequate intake, it will be reflected in her iron stores and later in haemoglobin concentration [6,7].

To overcome this problem, antenatal patients are supplemented with oral iron (60 mg of elemental iron/day)[8]. Oral iron is also used as the first line of treatment for iron deficiency anemia during pregnancy. However, due to various side effects such as abdominal discomfort, nausea, vomiting, constipation, and dark-colored stools, oral iron has low compliance [9,10,11,12].

Parenteral iron therapy is an attractive option for rapid replenishment of iron stores without the issue of compliance [13]. Previous parenteral preparations like iron dextran and iron sorbitol had high rates of serious adverse effects that included anaphylaxis and even death. However, the advent of newer parenteral iron preparation has revolutionized the treatment of IDA. Iron sucrose (FeS) is widely used nowadays for the treatment of IDA, as there are numerous studies proving its safety during pregnancy. However, the biggest drawback of FeS is that it can only be given in doses of 200 mg or 300 mg, repeated on alternate days, so multiple hospital visits are required for the completion of therapy. Recently, ferric carboxymaltose (FCM) was introduced, which can be given intravenously in doses of 500 mg or 1000 mg and repeated weekly if required. It has fewer side effects than FeS and requires fewer hospital visits. So, it should be used as first-line therapy for iron deficiency during the third trimester, where rapid replenishment is required [14].

There are studies reported in the literature which compared FCM and FeS in terms of safety and efficacy. Most of these studies have been done in postpartum women with anemia [15,16,17,18,19,20]. Only a few studies have been done in pregnant women, and most of these are retrospective [21,22]. We conducted a prospective study in antenatal women with moderate to severe IDA to compare the safety and efficacy of FCM and FeS at a tertiary care hospital.

## Methods

A prospective observational study was conducted in the Department of Obstetrics and Gynecology at a tertiary care hospital from September 2018 to June 2020. The study was started after approval from the Institutional Ethics Committee. Antenatal women between 14 and 36 weeks of gestation with moderate or severe iron deficiency anemia (Hb < 10 g%) who were either intolerant, non-compliant or not responding to oral iron were enrolled in the study. Women with anemia not linked to iron deficiency, allergy/hypersensitivity/intolerance of parenteral iron preparations, chronic liver disease, history of asthma, haemoglobin < 6 g/dL and subjects who had received a blood transfusion within last 3 months were excluded from the study. After careful history taking, clinical examination, and laboratory investigations, other causes of anemia were ruled out. Informed consent was obtained from all women. We also enquired about symptoms of anemia like lethargy, easy fatigability, irritability, pica, palpitation, or breathlessness. Subjects were counselled regarding two parenteral iron preparations, including the dosage, cost, side effects, and number of visits and were asked for their choice. They were then divided into two groups.

**Group I:** FCM was administered in a single, once weekly infusion of 1000 mg, diluted in 250 mL of 0.9% normal saline over 15–20 min on day 1 and, if needed, on day 8.

**Group II:** Total required intravenous FeS was given in divided doses of 200 mg, diluted in 100 mL of 0.9% normal saline on alternate days, over 15–20 min.

Total iron deficit was calculated according to Ganzoni’s formula[23]:

Body weight (kg) × 2.3 × (12 - patient’s haemoglobin, g/dL) + 500 mg (for stores).

A test dose was given before the therapy as a slow infusion over 4–5 min. If the patient did not show any reaction, the remaining drug was administered. Whole therapy was monitored, and each recipient was kept under observation in hospital for at least 2 h after administration of parenteral iron. The subjects were observed for signs of intolerance, such as anaphylactic reactions, skin rash, dyspnoea, facial flushing, urticaria, hypotension, headache, chest pain, tachycardia, breathlessness, etc. Any minor or major adverse or side effects were noted. Minor side effects included cannula site irritation, itching, flushing, skin rash, metallic taste, headache, and nausea. Major side effects included dyspnoea, hypotension, chest pain, tachycardia, anaphylactic reaction, and death. All subjects were called at 2 and 4 weeks after completion of therapy. The following tests were carried out at each visit: complete blood count, red cell indices including MCV, reticulocyte count, serum ferritin and total iron binding capacity (TIBC) on day 14 and day 28. Subjects were queried about improvement in symptoms at each visit. All other investigations were done as per need of the patient according to standard hospital protocol. Study participants were followed up until delivery for maternal and neonatal outcome.

### Statistical analysis

Sample size was calculated based on the difference in mean haemoglobin, as reported in previous studies [22], to achieve a statistically valid comparison of the two groups. With a type 1 error of 0.05, a power of 80%, and a 20% attrition rate, a sample size of at least 43 women in each group was required.

Data were presented as number (per cent)/mean ± SD (standard deviation) or median (interquartile range) as appropriate. Categorical variables were compared between the groups using the chi square/Fisher’s exact test, and continuous variables were compared using the student’s t-test. The variables that did not follow normal distribution were compared between the groups using the non-parametric Mann–Whitney U test. A *p* value of less than 0.05 was considered statistically significant. All the analysis was performed by using the Statistical Package for Social Sciences (SPSS) software 21.

## Results

One hundred and twenty-three antenatal women with moderate to severe iron deficiency anemia were approached for the study. Eight patients refused to give consent, four patients with severe anemia had had a blood transfusion in the last month, nine patients had received parenteral iron injections outside in the last 6 weeks, two patients had chronic liver disease, and five patients did not receive full treatment. A total of 95 were included in final analysis. Forty-nine patients received FCM, while 46 patients received FeS. In the FCM group, there were 41 patients with moderate anemia (Hb between 7 g/dl to 9.9 g/dl) and eight patients with severe anemia (Hb between 6 g/dl and 7 g/dl). There were 43 patients with moderate anemia (Hb between 7 g/dl and 9.9g/dl) and 3 patients with severe anemia (Hb between 6 g/dl and 7 g/dl) in the FeS group.

The demographic profile of the women in both the groups was comparable. The baseline characteristics of the study participants were also similar, as shown in Table 1.

**Table 1.** Baseline characteristics of study participants in the two groups.

**Characteristics**	**Group I (FCM)**	**Group II (FeS)**	***p* value**
Age (year)^*^	25.49 ± 4.63	25.36 ± 3.94	0.90
BMI (kg/m^2^)^*^	23.77 ± 3.55	24.54 ± 1.11	0.35
Gravidity^*^	1.89 ± 1.11	1.78 ± 0.98	0.63
Haemoglobin (g/dL)^*^	8.61 ± 0.87	8.55 ± 0.78	0.78
Serum ferritin (ng/mL)^**^	6.4 (1.8–25.9)	6.85 (0.6–16.9)	0.87
Serum iron (mcg/dL)^**^	25 (11–292)	17 (8–391)	0.06
MCV (fL)^*^	73.52 ± 9.05	73.08 ± 8.05	0.82
TIBC (mcg/dL)^*^	540.38 ± 99.55	562.17 ± 107.15	0.36
Reticulocyte count (%)^*^	2.01 ± 0.66	1.91 ± 0.58	0.46

* Data expressed as mean ± SD.

** Data expressed as median (interquartile range).

There was a significant rise in Hb after therapy in both groups. The rise in mean haemoglobin was significantly more in FeS group after 14 days as compared to the FCM group [1.69 ± 0.98 g/dL vs. 2.25 ± 0.91 g/dL, *p* value = 0.01]. But this rise was not significantly different after 28 days [2.50 ± 1.15 g/dL vs. 2.85 ± 1.18 g/dL, *p* value = 0.20]. The median rise in serum ferritin level was significantly higher in the FCM group as compared to the FeS group after 14 days and 28 days, as shown in Table 2 [*p* value < 0.001 and 0.001, respectively). A rise in MCV and reticulocyte count was comparable in both groups. TIBC showed a significant rise in group I as compared to group II after 28 days of therapy.

**Table 2.** Change in haematological indices after parenteral iron therapy.

**Variable**	**Group I (FCM)**	**Group II (FeS)**	***p* value**
Haemoglobin (g/dL)			
Rise after 14 days^*^	1.69 ± 0.98	2.25 ± 0.91	0.01
Rise after 28 days^*^	2.50 ± 1.15	2.85 ± 1.18	0.20
Serum ferritin (ng/mL)			
Rise after 14 days^**^	310.60 (89.20–1203.20)	148.55 (0.20–516.19)	< 0.001
Rise after 28 days^**^	94.10 (18.20–1035.40)	71.60 (0.10–473.85)	0.001
MCV (fL)			
Rise after 14 days^**^	8.2 (0.0–24.2)	9.8 (3.1–23.47)	0.10
Rise after 28 days^**^	10.1 (0.60–26.8)	11.2 (0.0–21.27)	0.71
Reticulocyte count (%)			
Rise after 14 days^**^	0.82 (0.00–3.89)	0.91 (0.00–2.63)	0.65
Rise after 28 days^**^	0.86 (0.01–2.25)	0.72 (0.06–1.80)	0.27
TIBC (mcg/dL)			
Fall after 14 days^**^	133.0 (5–307)	99 (7–254)	0.20
Fall after 28 days^**^	192 (17–411)	139 (23–336)	0.05

* Data expressed as mean ± SD.

** Data expressed as median (interquartile range).

Most of the subjects were asymptomatic at presentation. Out of 42 subjects with symptoms, 23 were in group I and 19 were in group II. In group I, 15 (65.21%) subjects and in group II, 15 (78.95%) subjects had improvement in symptoms within 1 week after therapy. Seven (30.43%) subjects in group I and four (21.05%) subjects in group II had improvement of symptoms within 2 weeks. One (4.35%) patient in group I showed improvement in 3 weeks. No major side effects were observed in the two groups after iron therapy (Table 3). However, three subjects in group II had minor side effects, of which, one patient had cannula site irritation, one had rashes all over the body, and one patient had nausea.

**Table 3.** Comparison of maternal and neonatal outcomes in the two groups.

	**Group I (FCM)**	**Group II (FeS)**
Total patients with symptoms	n = 23 (%)	n = 19 (%)
Improvement in 1 week	15 (65.21)	15 (78.95)
Improvement in 2 weeks	7 (30.43)	4 (21.05)
Improvement in 3 weeks	1 (4.35)	0
Side effects		
Major side effects	0	0
Minor side effects	0	3 (6.5)
Gestational age at delivery		
Term (37–42 weeks)	47 (95.9)	46 (100)
Preterm (< 37 weeks)	1 (2.04)	0
Postterm (> 42 weeks)	1 (2.04)	0
Birth weight		
Low birth weight (< 2.5kg)	1 (2.04)	3 (6.53)
Normal birth weight (2.5–4kg)	48 (97.96)	42 (91.3)
Macrosomia (> 4kg)	0	1 (2.18)
NICU admission		
Required	2 (4.08)	2 (4.34)
Not required	47 (95.91)	44 (95.65)

Neonatal outcomes in both the groups were comparable. There was no significant difference in the gestational age at delivery, birth weight, or NICU admission (Table 3).

## Discussion

FCM is reported to be safer and more cost effective for the treatment of iron deficiency anemia, as it can be administered in a single dose and with fewer adverse events [15,16,17,18,19,20,21,22,23,24]. In our study, we compared two intravenous iron preparations (FCM and FeS) for the treatment of iron deficiency anemia during the second and third trimesters of pregnancy. It was found that the rise in haemoglobin was higher in the FeS group after 14 days of therapy, but it became comparable after 28 days. The plausible explanation for the higher rise in FeS group in our study is that the Hb rise started after the first dose of FeS but was measured at completion of therapy, which took 10 to 12 days more than FCM. We also found that Inj FCM led to rapid replenishment of iron stores, as evident from the higher rise in serum ferritin as compared with FeS.

Inj FeS has been used for treatment of iron deficiency anemia for a long time, but the main disadvantages are the limited permissible dose per day, leading to the higher number of required visits. FCM, on the other hand, can be administered in higher doses, thus requiring a fewer number of visits. People have reported its higher efficacy in the postpartum period as compared to FeS. It is more beneficial in such postpartum women as a hospital stay is not prolonged due to FCM administration in a single dose, while for FeS, either prolonged hospitalization or repeated visits are required to complete therapy. So, FCM has been the treatment of choice in postpartum women with iron deficiency anemia [15,17,19,20,21,22,23,24]. Only a few studies have compared FCM and FeS during pregnancy.

The first study comparing Inj FCM and Inj FeS for correction of iron deficiency anemia was conducted by Cristoph et al. in 2012 [21]. They compared the rise in Hb after 28.4 days of treatment with FCM and 41.2 days after FeS therapy and found that it was comparable in both groups, as in our study. They also concluded that both the drugs were safe during pregnancy. This study also considered more time taken for completion of therapy. So, Hb levels were done at 41.2 days in the FeS group.

Rathod et al., in a randomised prospective study in antenatal and postpartum women, compared FCM, FeS, and oral iron [15]. A significantly greater rise in haemoglobin and fewer adverse effects were documented with FCM as compared to FeS and oral iron. Similar results were obtained by Patel et al. in a prospective observational study comparing FCM and FeS in pregnant as well as postpartum subjects [17]. Boughton et al., in a retrospective audit in pregnant women between 2007 and 2013, found that FCM had an advantage over FeS, as it offered a higher rise in haemoglobin level within a very short interval of time, and with a comparable neonatal outcome [22]. In that study, women were followed for gestational age at delivery, and most of the women delivered at term in both groups; birth weight was also average for gestational age for most of the babies in both groups.

Jose et al. conducted a randomised clinical trial in a tertiary care hospital and showed similar replenishment of iron stores with both FCM and FeS therapy, but FCM led to an early and higher rise in haemoglobin, which contrasts with the present study [24]. So, FCM can be considered for women nearer to term.

Singh et al. and Lunagariya et al. conducted a prospective study in postpartum women and concluded that FCM had a better safety profile and resulted in faster rise in haemoglobin and iron stores as compared to FeS [19,20]. FCM had the added benefit of a shorter duration of hospital stay. Hol et al. and Qassim et al. concluded that both drugs had similar efficacy and safety for the treatment of iron deficiency anemia [18,25]. Another recent study done over a six-year period by Patel et al. also concluded that FCM results in a higher rise in Hb and serum ferritin and hence is better than FeS [26]. According to that study, done with 334 patients, both the iron preparations are well tolerated during pregnancy [26]. So, the choice is mainly determined by cost effectiveness and the convenience of the recipient.

The strength of this study was that it was a prospective study in pregnant women at a tertiary centre. The subjects were properly followed up after iron injections, which allowed for proper documentation of efficacy and side effects. This was done in pregnant women, while most of the previous studies had been done in postpartum or gynaecological subjects.

The limitation of our study was that it was an observational study and not a randomised trial, and it had a limited sample size. Larger randomised trials are required to generate further data on the safety and efficacy of the two iron preparations in pregnant women.

It was concluded from the study that there is comparable rise in haemoglobin after 1 month of FCM and FeS therapy, while FCM resulted in earlier replenishment of body iron stores as compared to FeS, which is reflected by a higher rise in serum ferritin levels. The two iron preparations are safe and comparable in terms of major side effects. Our study indicates that pregnant women can be treated effectively by FCM with the advantage of a single infusion, fewer side effects, and better compliance. It reduces the number of visits to hospital, thereby reducing bed occupancy rate and the burden on health facilities in developing countries.
